# The Added Value of Remote Technology in Cardiac Rehabilitation on Physical Function, Anthropometrics, and Quality of Life: Cluster Randomized Controlled Trial

**DOI:** 10.2196/42455

**Published:** 2023-04-12

**Authors:** Heli Lahtio, Ari Heinonen, Teemu Paajanen, Tuulikki Sjögren

**Affiliations:** 1 Faculty of Sport and Health Sciences University of Jyväskylä Jyväskylä Finland; 2 LAB University of Applied Sciences Lahti Finland; 3 Finnish Institute for Health and Welfare Helsinki Finland

**Keywords:** weight loss, cardiac rehabilitation, remote technology, physical function, 6-minute walk test, overweight, obesity, body mass, BMI, waist circumference, quality of life, QoL, mobile phone

## Abstract

**Background:**

Cardiovascular diseases (CVDs) cause most deaths globally and can reduce quality of life (QoL) of rehabilitees with cardiac disease. The risk factors of CVDs are physical inactivity and increased BMI. With physical activity, it is possible to prevent CVDs, improve QoL, and help maintain a healthy body mass. Current literature shows the possibilities of digitalization and advanced technology in supporting independent self-rehabilitation. However, the interpretation of the results is complicated owing to the studies’ high heterogeneity. In addition, the added value of this technology has not been studied well, especially in cardiac rehabilitation.

**Objective:**

We aimed to examine the effectiveness of added remote technology in cardiac rehabilitation on physical function, anthropometrics, and QoL in rehabilitees with CVD compared with conventional rehabilitation.

**Methods:**

Rehabilitees were cluster randomized into 3 remote technology intervention groups (n=29) and 3 reference groups (n=30). The reference group received conventional cardiac rehabilitation, and the remote technology intervention group received conventional cardiac rehabilitation with added remote technology, namely, the Movendos mCoach app and Fitbit charge accelerometer. The 12 months of rehabilitation consisted of three 5-day in-rehabilitation periods in the rehabilitation center. Between these periods were two 6-month self-rehabilitation periods. Outcome measurements included the 6-minute walk test, body mass, BMI, waist circumference, and World Health Organization QoL-BREF questionnaire at baseline and at 6 and 12 months. Between-group differences were assessed using 2-tailed *t* tests and Mann-Whitney *U* test. Within-group differences were analyzed using a paired samples *t* test or Wilcoxon signed-rank test.

**Results:**

Overall, 59 rehabilitees aged 41 to 66 years (mean age 60, SD 6 years; n=48, 81% men) were included in the study. Decrement in waist circumference (6 months: 1.6 cm; *P*=.04; 12 months: 3 cm; *P*<.001) and increment in self-assessed QoL were greater (environmental factors: 0.5; *P*=.02) in the remote technology intervention group than the reference group. Both groups achieved statistically significant improvements in the 6-minute walk test in both time frames (*P*=.01-.03). Additionally, the remote technology intervention group achieved statistically significant changes in the environmental domain at 0-6 months (*P*=.03) and waist circumference at both time frames (*P*=.01), and reference group achieve statistically significant changes in waist circumference at 0-6 months (*P*=.02).

**Conclusions:**

Remote cardiac rehabilitation added value to conventional cardiac rehabilitation in terms of waist circumference and QoL. The results were clinically small, but the findings suggest that adding remote technology to cardiac rehabilitation may increase beneficial health outcomes. There was some level of systematic error during rehabilitation intervention, and the sample size was relatively small. Therefore, care must be taken when generalizing the study results beyond the target population. To confirm assumptions of the added value of remote technology in rehabilitation interventions, more studies involving different rehabilitees with cardiac disease are required.

**Trial Registration:**

ISRCTN Registry ISRCTN61225589; https://www.isrctn.com/ISRCTN61225589

## Introduction

### Background

Technology-based rehabilitation with physical activity has been widely studied in rehabilitees with several diseases [1,2]. Cardiovascular diseases (CVDs) are the leading cause of death worldwide. They caused >30% of deaths in 2019 [3]; furthermore, heart diseases cause approximately 16% of deaths globally [4]. However, the effectiveness of technology-based rehabilitation and the added value of technology-assisted home-based self-rehabilitation has not been widely studied in rehabilitees with cardiac disease in rehabilitation settings. Self-rehabilitation is planned with the consultation of health care professionals and considers the needs of rehabilitees under rehabilitation as well as evidence-based practices [5].

Telehealth- (telephone, computer, internet, and videoconferencing) [6] and smartphone-based [7] cardiac rehabilitation has been found to be as effective as center-based rehabilitation in decreasing coronary risk factors [6,7]. The best-known evidence-based practices are traditionally based on minimizing CVD risk factors, such as high blood pressure, physical inactivity, unhealthy diet, tobacco consumption, and harmful use of alcohol [3,8], as well as increased BMI [9-11]. Increasing moderate physical exercise reduces the risk of CVDs and their symptoms [12]. In cardiac rehabilitation, rehabilitees should undertake regular physical activity that slows the progression of CVDs [13]. Physical activity can prevent CVDs and improve quality of life (QoL) [14].

The current goal of rehabilitation is to support and maintain individuals’ physical, mental, and social resources [15] and to maintain or achieve a healthier lifestyle and well-being using mixed methods of face-to-face rehabilitation and self-rehabilitation using remote technology [16,17]. The current literature shows that digitalization and advanced technology can support independent home-based rehabilitation in different rehabilitation groups [1,18-21] and improve physical activity [22] and body composition [23]. Technology-based interventions may also improve lifestyle risk factors and disease management in rehabilitees with cardiac diseases [24-26]. In addition, mobile technology can increase cardiac rehabilitation adherence [27] and compliance [28].

There is a lack of specific knowledge regarding the effectiveness or added value of remote technology-based rehabilitation in rehabilitees with CVD. To the best of our knowledge, 2 studies have examined the added value of digital health interventions and yielded contradictory results [29,30] on body composition compared with conventional cardiac rehabilitation. Widmer et al [29] found that smartphone- and web-based groups achieved statistically significant improvements in weight loss compared with usual care, but Pfaeffli Dale et al [30] did not find statistically significant differences between the mobile health and usual care groups in weight, BMI, or waist-to-hip ratio.

### Objective

In this study, a biopsychosocial perspective on technology-based self-rehabilitation was considered. This study aimed to investigate at the individual level the added value of remote technology in cardiac rehabilitation on physical function, anthropometrics, and QoL in participants with CVD compared with conventional rehabilitation. Rehabilitees attended standard group-based cardiac rehabilitation courses at a rehabilitation center in Finland. Because of the possibility of cross-contamination between the experimental and control groups, cluster randomization was performed.

## Methods

### Study Design and Randomization

This study (registration number ISRCTN61225589) was a cluster randomized trial. Recruitment and data collection were conducted between September 2015 and May 2017 at the Finnish Rehabilitation Center. This study was a real-life research project with a 1-year data recruitment period.

Rehabilitees were distributed into 6 groups by officers of the Social Insurance Institution of Finland. Randomization occurred at the group level in pairs of 2 consecutive groups with following two experimental arms: (1) conventional cardiac rehabilitation with remote technology (remote technology intervention) group and (2) conventional cardiac rehabilitation (reference) group. The rehabilitees were randomized into 3 remote technology intervention clusters and 3 reference clusters. Rehabilitees were cluster randomized into cluster pairs, which bypassed any systematic bias that occurred because of the season. The clusters began in autumn (September to November), winter (December to February), and spring (March to May). The other cluster randomization–specific confounding factors, such as department and caregiver factors, were controlled in the study design using the same rehabilitation center and caregivers in all remote technology interventions (n=3) and reference groups (n=3).

Randomization was accomplished with sealed envelopes within rehabilitation groups, and a person outside the research group (researcher of the gerontological research center) processed the randomization under the supervision of 2 researchers (TS and Heikki Kivistö). Randomization was performed 3 times for 2 consecutive groups, considering the season and months. In total, 59 rehabilitees were randomly assigned in pairs into (1) the remote technology intervention group (n=29) or (2) the reference group (n=30) in consecutive order. At the beginning of the rehabilitation, the researchers (TS and HK) informed the rehabilitees about the group (n=6) their rehabilitation group had been randomized. After randomization, rehabilitees provided written consent for their participation in the intervention. Because of the nature of the intervention, it was not possible to blind the rehabilitees and caregivers in terms of the intervention. The outcome assessor was not blinded to the intervention, but one educated person who was not involved in the study group performed the anthropometric measurements, and another person performed the 6-minute walk tests (6MWTs). The statistician was blinded to the interventions.

**Figure 1:**
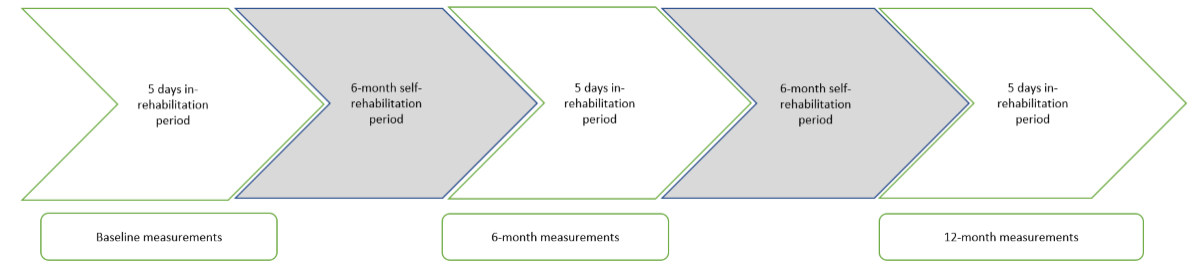
The design of the cardiac rehabilitation study.

Cardiac rehabilitation was implemented in groups of rehabilitees. The rehabilitees attended 3 sessions during the 5-day in-rehabilitation period in the rehabilitation center. Between these rehabilitation periods, there were two 6-month self-rehabilitation phases. All outcome measurements were performed at baseline and at the 6- and 12-month follow-ups (Figure 1).

### Ethics Approval

The study was approved on October 15, 2015, by the Ethics Committee of the Central Finland Health Care District (Dnro: 12 U/2015).

### Rehabilitees

A total of 59 rehabilitees within the 6 rehabilitation clusters were recruited among the rehabilitees attending coronary heart disease rehabilitation courses between September 2015 and May 2017. The rehabilitees applied for the course with a physician’s referral. The primary eligibility criterion for the study was an adult (aged <18 years) with coronary heart disease and having independent basic level of management of IT and remote technology applications. The exclusion criteria included having serious musculoskeletal disorders, cognitive diseases, and memory diseases that affect essential functional abilities to ensure the capability of rehabilitees to use technology. The inclusion and exclusion criteria were based on functional ability because this study is interested in the added value of technology in cardiac rehabilitation, regardless of the background of the rehabilitees, for example, medication or comorbidities. All rehabilitees who underwent cardiac rehabilitation fulfilled the inclusion criteria. There were no statistically significant differences in the demographic and clinical characteristics at baseline between the remote technology intervention and reference groups (Table 1).

The risk of comorbidities and cointerventions was evaluated before the rehabilitation intervention using a baseline questionnaire. The number and type of comorbidities were assessed in the questionnaire (Table 1). The risk of cointervention was evaluated by asking whether the rehabilitees had made lifestyle changes during the previous 12 months. There were no statistically significant differences in the number of comorbidities (*P*=.65) at baseline and only minor lifestyle changes were observed before the intervention. Of the 59 rehabilitees, 8 (14%) reported changing their lifestyle to a healthier direction during the last 12 months. Rehabilitees reported minor changes, such as eating more vegetables (n=4), changing the quality or amount of fat (n=2), decreasing salt use (n=1), or losing weight (n=1; Table 1). In Finland, it is possible to participate in only 1 rehabilitation intervention at the same time as the Social Insurance Institute of Finland affirmed rehabilitation interventions. During the intervention, the rehabilitees had normal health care services, if needed. In addition to questions regarding comorbidities and lifestyle changes, the baseline questionnaire included questions regarding lifestyle habits, such as what kind of fat rehabilitees used on bread and in cooking. There was also a question regarding what kind of milk the rehabilitees used and how much alcohol they used.

The mean age of the rehabilitees was 60 (SD 6; range 41-66) years, and 81% (48/59) of them were men. Rehabilitees underwent coronary angioplasty or coronary artery bypass 3-12 months before the intervention.

**Table 1:** Baseline demographic and clinical characteristics of the rehabilitees.

			Remote technology intervention group (n=30)		Reference group (n=29)	*P* value
Age (years), mean (SD)			59.7 (6)		59.2 (6.1)	.64
**Sex, n (%)**						
	Female	7 (23)		4 (14)		
	Male	23 (77)		25 (86)		
Baseline body mass (kg), mean (SD)			86.9 (19.1)		86.7 (16.2)	.34
Baseline BMI (kg/m^2^), mean (SD)			29 (5.2)		28.5 (4)	.37
Baseline waist circumference (cm), mean (SD)			103.7 (12.9)		103.5 (11.6)	.61
Number of comorbidities, mean (SD)			3.8 (1.2)		3.3 (1.1)	.21
**Number of angioplasties, n (%)**			30 (100)		27 (100)	
	0-1	25 (83)		24 (89)		
	2-3	4 (13)		2 (7)		
	4-6	1 (3)		1 (4)		
**Bypass surgery, n (%)**						
	Yes	7 (23)		2 (7)		
	No	23 (77)		25 (93)		
**Myocardial infarction, n (%)**						
	Yes	11 (37)		19 (68)		
	No	18 (60)		7 (23)		
**Time of the operation (months), n (%)**			30 (100)		26 (100)	
	0-3	3 (10)		3 (11)		
	3-6	2 (7)		8 (29)		
	6-12	14 (47)		10 (36)		
	>12	10 (33)		4 (14)		
	During the rehabilitation	1 (3)		1 (4)		
**Education, n (%)**			24 (100)		28 (100)	
	No occupational education	5 (21)		3 (11)		
	Course-based education	1 (4)		4 (14)		
	Vocational education	13 (54)		9 (32)		
	Community college	2 (8)		6 (21)		
	Higher education	2 (8)		5 (18)		
	Other	1 (4)		1 (4)		
**Working status, n (%)**			23 (100)		28 (100)	
	Full-time job	14 (61)		11 (39)		
	Part-time job or part-time retired	1 (4)		2 (7)		
	Unemployed, laid off, on long-term sick leave, or rehabilitation support	2 (9)		6 (21)		
	Retired	4 (17)		4 (14)		
	Other	2 (9)		5 (18)		
**Comorbidities, n (%)**			30 (100)		29 (100)	
	High blood pressure	14 (47)		15 (52)		
	High blood cholesterol	14 (47)		20 (69)		
	Diabetes	1 (3)		7 (24)		
	Heart attach	11 (37)		13 (45)		
	Coronary artery disease or angina pectoris	24 (80)		21 (72)		
	Cancer	0 (0)		0 (0)		
	Rheumatoid arthritis	1 (3)		2 (7)		
	Back disease	8 (27)		8 (28)		
	Chronic bronchitis	2 (7)		0 (0)		
	Depression	3 (10)		1 (3)		
	Other mental problem	1 (3)		1 (3)		
	Asthma	2 (7)		3 (10)		
	Stomach disease (eg, gastritis)	1 (3)		1 (3)		
**Eating habits (points), mean (SD)**			29 (100)		28 (100)	
	What kind of fat you use on a bread?	3.8 (2)		3.1 (1.7)		.15
	What kind of fat you use in cooking?	3.3 (2.7)		3.1 (2.8)		.85
	What kind of milk you use?	4.8 (1.4)		4.7 (1.2)		.74
	How often you drink alcohol?	4.1 (1.2)		4 (1.3)		.67

### Intervention

Cardiac rehabilitation courses were held at the Finnish Rehabilitation Center and arranged by the Social Insurance Institution of Finland. The aim of conventional Finnish cardiac rehabilitation courses was to promote the biopsychosocial functional ability of rehabilitees and their ability to work [31]. The total duration of the cardiac rehabilitation intervention was 12 months. In cardiac rehabilitation, evidence-based rehabilitation methods and Finnish guidelines [15] were used.

### Conventional Cardiac Rehabilitation

Both the remote technology intervention and reference groups received conventional cardiac rehabilitation. The initiation of the intervention included a 5-day in-rehabilitation period. Furthermore, 2 follow-up periods of 5 days at the 6- and 12-month time points in the rehabilitation center were included in the study (Figure 1). Between these periods, the rehabilitees followed a 6-month home exercise program. At the rehabilitation center, conventional cardiac in-rehabilitation consisted of multidisciplinary rehabilitation; medical examination; physiotherapy; aqua therapy; gym training; stretching; aerobic exercises; and group discussions with a nutritionist, social worker, physiotherapist, psychologist, and physician. Rehabilitees received information and pamphlets regarding CVDs and the management of daily activities, such as dietary habits, relaxation, physical activity, social security benefits, self-care, and self-rehabilitation while living with CVDs. In addition, they underwent various tests for different health-related functions. During the in-rehabilitation periods (baseline and 6 and 12 months), the researcher (HK) provided group-based feedback on physical activity in group discussions to the remote technology intervention and reference groups. After the intervention, the rehabilitees received personal information on physical activity and other outcomes on a paper form.

The researchers at the University of Jyväskylä, Finland, were in equal contact with the rehabilitees in the remote technology intervention and reference groups during the different phases of the study. Furthermore, intervention, initiation, and follow-up periods in the remote technology intervention and reference groups were equal in the time sequence of the rehabilitation stages.

### Reference Group

The rehabilitees in the reference group only received printed materials for conventional cardiac rehabilitation. They did not have any mobile phone apps or other remote technology, but the reference group had standard phone communication with rehabilitation staff if needed. In the reference group, self-monitoring was performed using pen and paper.

### Remote Technology Intervention Group

At baseline, during the initiation of the 5-day in-rehabilitation period in the rehabilitation center, the remote technology intervention group received instructions on how to use the Movendos mCoach internet software (Movendos Ltd) via a mobile phone, computer, or tablet computer. Movendos mCoach is a browser-based tool that enables communication between the rehabilitee and instructor and monitors the progress of rehabilitee. Wrist-worn Fitbit Charge HR (Fitbit) accelerometers were used for self-monitoring and motivating physical activation during the 12-month intervention period. The accelerometers were guided to be worn daily and to follow the number of steps and other parameters describing physical activity. If required, the rehabilitees had an opportunity to use the Fitbit app, which described graphically and cumulatively reported daily, weekly, and monthly activity levels.

During the in-rehabilitation periods, the rehabilitees used the Movendos mCoach app, for example, in goal setting and exercise instructions, and Fitbit accelerometers for self-monitoring of physical exercises. In addition, the accelerometer serves as a motivational tool. In the remote technology intervention group, the remote technology application was used in home exercise instructions, advice, and monitoring of the rehabilitees during the 5-day in-rehabilitation and 6-month self-rehabilitation periods. Researchers and health care professionals did not receive real-time feedback from the accelerometers.

During the self-rehabilitation period, the remote technology intervention group performed monthly tasks that aimed to increase participants’ ability to cope with cardiac illness in everyday life. Web-based software (Movendos) sent monthly automatic motivational messages. In addition, peer support was possible in group discussions. The coaching software also allowed for changing experiences with the peer group. If required, the rehabilitees formed WhatsApp (Meta) groups, but this was not part of official rehabilitation. There was also the possibility of sending messages to the physiotherapist through coaching software. In addition, rehabilitees had an opportunity to contact the researcher responsible for measurement and technical support in problems related to the use of technology, if needed.

### Outcome Measurements

The primary outcome of this research project was a physical activity assessment published by Hakala et al [22]. This secondary study used individually measured outcomes, which were chosen based on the following biopsychosocial model: the 6MWT, BMI, waist circumference, and the World Health Organization QoL-BREF (WHOQOL-BREF) questionnaire. Measurements were performed at the rehabilitation center at baseline and at the 6- and 12-month measurement points. Anthropometrics were measured by a nurse, 6MWTs were measure by physiotherapists, and the WHOQOL-BREF questionnaires were measured by the researchers.

### Physical Function

Physical function was evaluated using the 6MWT. In the 6MWT, a rehabilitee walked at a self-selected pace for a 6-minute period. The rehabilitees were allowed to stop if needed and continue walking when they felt comfortable doing so [32,33]. The distance walked, heart rate, and symptoms of rehabilitees were recorded. The 6MWT is a valid and reliable field test [32,34].

### Anthropometry

Body mass was measured using a calibrated floor scale. The rehabilitees wore light clothes, such as T-shirts and shorts or tights, and shoes were off. BMI was calculated by dividing the weight by the square of the height in meters [35]. Waist circumference was measured on bare skin from the midpoint of the lowest rib to the iliac crest [36,37]. Overweight and obesity are defined at the population level as a BMI ≥25 kg/m^2^ and >30 kg/m^2^ [35], respectively. According to the World Health Organization, in 2016, approximately 39% of adults globally were overweight and 13% were reported to be obese [35]. It has been estimated that approximately 80% of people with coronary heart disease are overweight or obese [38]. They are also at a high risk of having a reduced QoL [13,39], and they require structured support to regain their QoL [40].

### QoL Measurement

QoL was measured using the WHOQOL-BREF questionnaire. The researchers were responsible for completing the questionnaires at the rehabilitation center in paper format. The questionnaire has 26 questions, one of which is related to general health and the other to overall QoL. The other 24 questions are related to 4 different domains: physical (7 items), psychological (6 items), social (3 items), and environmental (8 items). Higher scores reflected higher QoL. The WHOQOL-BREF has been translated into several languages, such as Finnish, German, and Japanese [40], and the Finnish version was used in this study.

### Statistical Analyses

Changes between the remote technology intervention and reference groups were compared between baseline and month 6 and between baseline and month 12. The changes in the measurement results were obtained by subtracting the results of the previous measurements from those of the previous measurements. Because of missing observations, testing was always performed by testing one time frame at a time, not all time frames at once. This approach provided an opportunity to test periods other than consecutive periods, for example, from baseline to 12 months.

Between-group differences were assessed using 2-tailed *t* tests and Mann-Whitney *U* tests. The normality of the groups had to be tested because the sample size in both groups was ˂50. Normality was tested using the Shapiro-Wilk test. If the *P* value was >.05, an independent samples *t* test was performed; otherwise, the Mann-Whitney *U* test was used. Variance in group equality was tested using Levene test. This was used in the case of the independent samples *t* test. If the *P* value was ˂.05 Levene test, 1-way ANOVA was performed. Otherwise, the variances were assumed to be equal and 2-way ANOVA was performed. Changes within groups were analyzed using a paired samples *t* test or Wilcoxon signed-rank test. In addition, between-clusters (1-6) differences were tested using 1-way ANOVA and Tukey test (0-6 and 0-12 months).

## Results

### Overview

**Figure 2:**
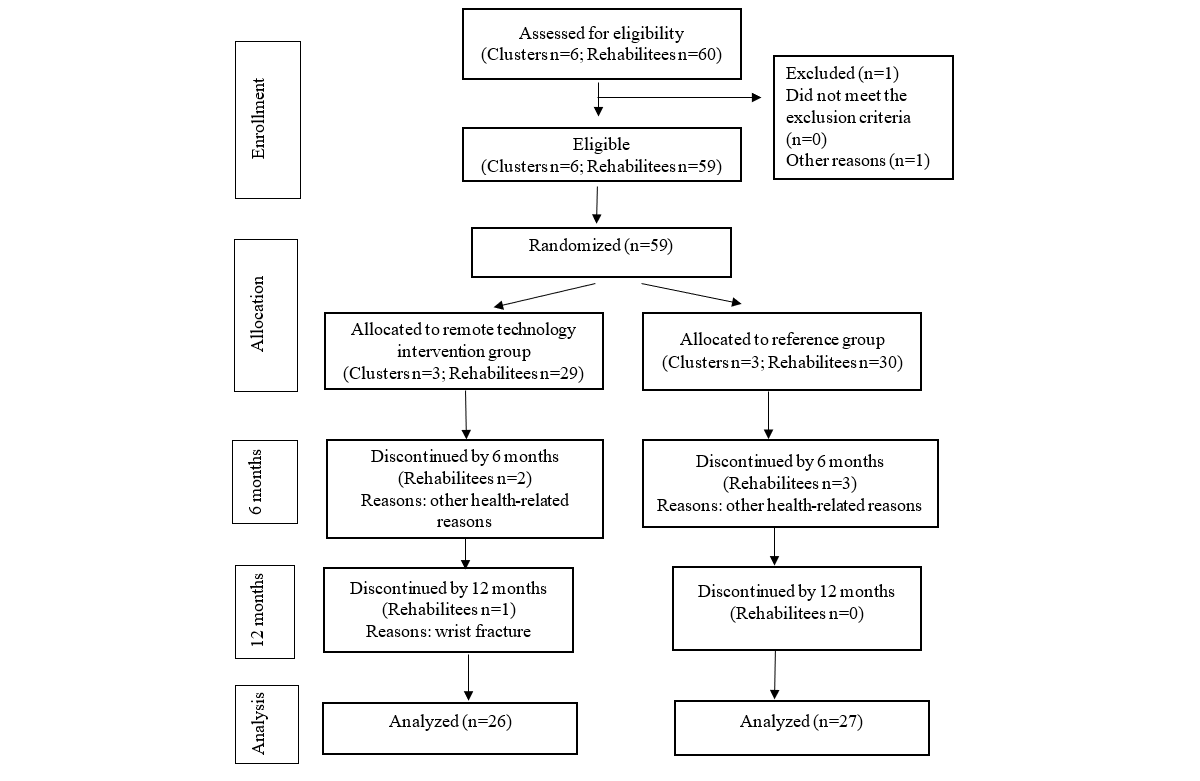
Flowchart of the intervention.

Among the 59 rehabilitees, the dropout rate was 10% over the 12-month period (Figure 2). At the 6-month follow-up point, 2 and 3 rehabilitees dropped out of the remote technology intervention and reference groups, respectively. At the 12-month follow-up, only 1 rehabilitee from the remote technology intervention group withdrew from the study. No serious adverse events occurred during the study. In the baseline questionnaire, statistically significant difference was reported regarding the fat content of bread. The mean value in the remote technology intervention group was 3.8 (SD 2) and in the reference group was 3.1 (SD 1.7; *P*=.02). At baseline, the remote technology intervention group used unhealthier fat than did the reference group. However, for other diet-related questions, there were no statistically significant between-group differences.

### Outcome Measurements

The waist circumference decreased significantly more at the 6-month (1.6 cm; *P*=.04) and 12-month (3 cm; *P*<.001) follow-up points in the remote technology intervention group than at those in the reference group (Table 2). Regarding the QoL, the environmental domain improved more in the remote technology intervention group than in the reference group (0.5; *P*=.02). There were no statistically significant differences in the other outcomes. In the between-cluster difference analysis, there were no statistically significant differences in any outcome.

**Figure 3:**
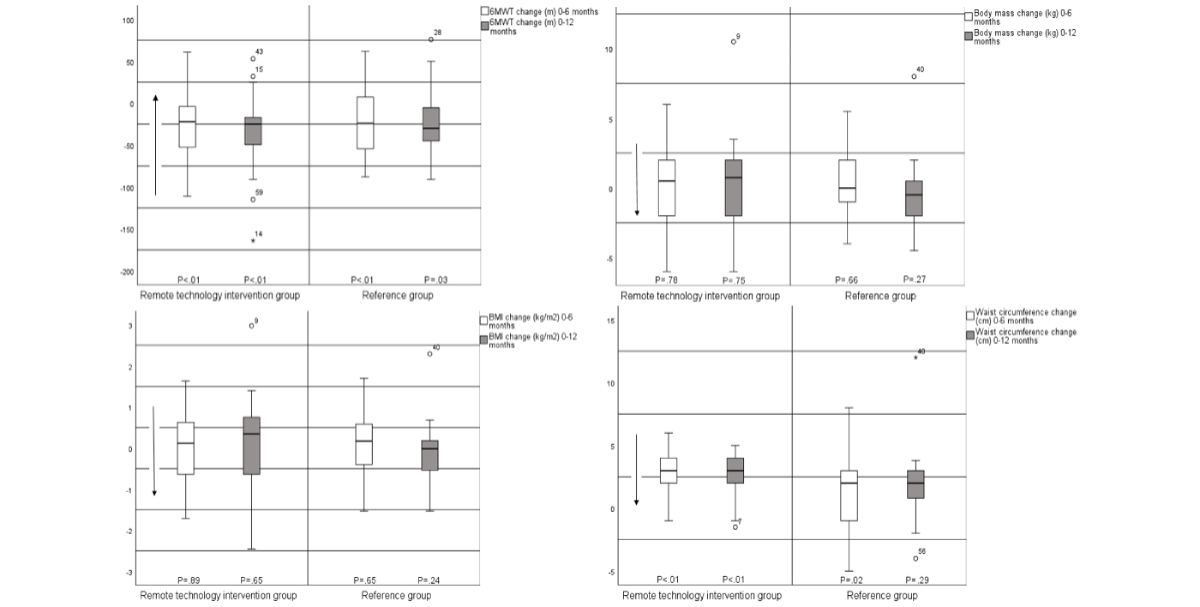
Within-group differences (median and IQR) in 6-minute walk test (6MWT), body mass, BMI, and waist circumference.

**Figure 4:**
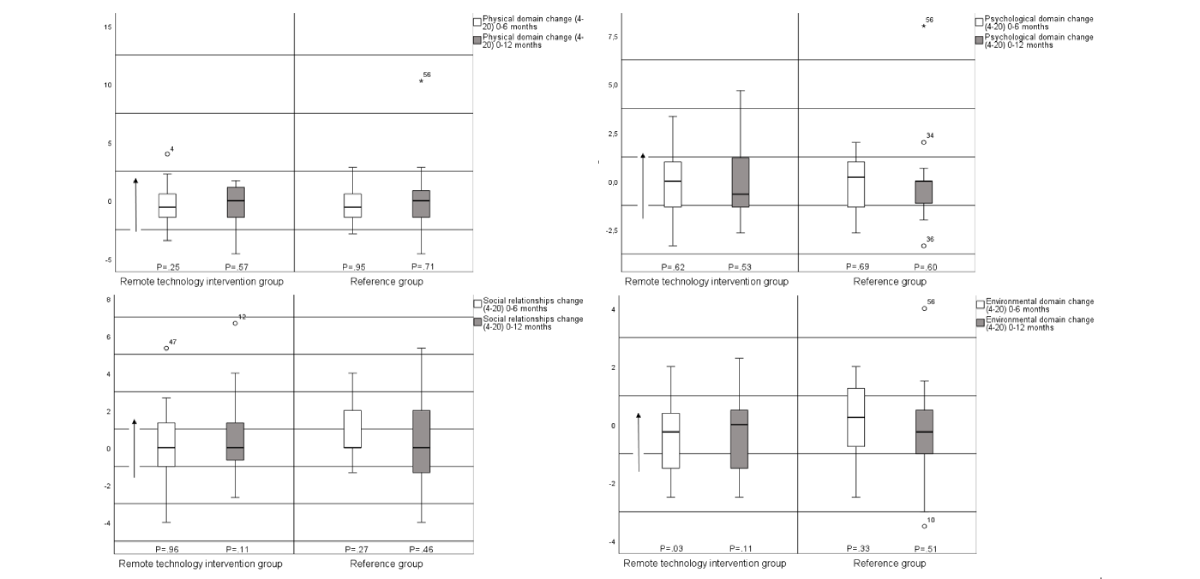
Within-group differences (median and IQR) in World Health Organization quality of life-BREF (WHOQOL-BREF; physical domain, psychological domain, social relationships, and environmental domain).

In the within-group analyses, statistically significant changes in waist circumference were seen in both groups at 0-6 months (intervention: *P*=.01; reference: *P*=.02) and in the remote technology intervention group at 0-12 months (*P*=.01; Figures 3 and 4 and Table 3). In addition, the 6MWT in both groups and both time frames achieved statistically significant changes (intervention: *P*=.01 for both; reference: 0-6 months, *P*=.03 and 0-12 months, *P*=.01), as did the environmental domain in the remote technology intervention group at 0-6 months (*P*=.03; Figures 3 and 4 and Table 3).

**Table 2:** Between-group differences of 6-minute walk test (6MWT), body mass, BMI, waist circumference, and World Health Organization quality of life (QoL)-BREF in the remote technology intervention and reference groups.

		Baseline, mean (SD)		Change to month 6, mean (SD; 95% CI)				Change to month 12, mean (SD; 95% CI)			
		Remote technology intervention (n=28)	Reference (n=29)	Remote technology intervention (n=26)		Reference (n=25)	*P* value	Remote technology intervention (n=24)		Reference (n=25)	*P* value
6MWT^a,b^ (m)		610 (68.1)	664 (84.2)	28.6 (44.3; −17.2 to 33.8)		20.3 (42.5; −17.2 to 33.8)	.52	32.9 (48.6; −17.4 to 37.2)		23.1 (40.5; −17.4 to 36.9)	.47
Body mass^c^ (kg)		86.9 (19.2)	86.7 (16.2)	−0.2 (2.8; −1.4 to 1.5)		−0.2 (2.2; −1.4 to 1.5)	.95	−0.2 (3.4; −2.5 to 1)		0.6 (2.5; −2.5 to 0.9)	.97
BMI^c^ (kg/m^2^)		29 (5.2)	28.5 (4.3)	−0.03 (0.9; −0.4 to 0.5)		0.07 (0.8; −0.4 to 0.5)	.25	−0.1 (1.2; −0.9 to 0.3)		0.2 (0.8; −0.9 to 0.3)	.69
Waist circumference (cm)		103.7 (13)	103.5 (11.6)	−3.1 (2.6; −3.2 to −0.1)		−1.5 (3; −3.2 to 0.1)	.04	−3 (3.1; −4.2 to −0.3)>		0.8 (3.7; −4.2 to 0.3)	<.001
**QoL^d^**											
	Physical (4-20)	14 (2.3)	14 (2.7)	0.4 (1.7; −0.8 to 1.5)		0.03 (2.1; −0.7 to 1.4)	.52	0.2 (1.6; −1 to 1.8)		−0.2 (2.9; −0.9 to 1.8)	.54
	Psychological (4-20)	15 (2.2)	14.8 (2.6)	0.2 (1.6; −0.6 to 1.1)		−0.1 (1.3; −0.6 to 1.1)	.53	0.2 (1.8; −1.2 to 1.2)		0.3 (2.2; −1.2 to 1.2)	.97
	Social relationships (4-20)	15.9 (2.8)	14.3 (3.8)	0.02 (2.2; −0.7 to 1.9)		0.5 (2.2; −0.7 to 1.9)	.39	−0.7 (2.1; −1.7 to 0.9)		−0.4 (2.2; −1.7 to 0.9)	.58
	Environmental (4-20)	14.6 (2.2)	14.9 (2.1)	0.5 (1.2; 0.1 to 1.5)		−0.3 (1.2; 0.1 to 1.5)	.02	0.5 (1.4; −0.7 to 1.1)		0.2 (1.6; −0.6 to 1.1)	.59

^a^6WMT: 6-minute walk test.

^b^Differences in sample size: 6MWT remote technology intervention group n=22; reference group n=25.

^c^Body mass and BMI: remote technology intervention group n=23; reference group n=26.

^d^QoL: remote technology intervention group n=24; reference group n=22.

**Table 3:** Within-group changes in the 6-minute walk test (6MWT), body mass, BMI, waist circumference, and quality of life (QoL).

					0-6 months							0-12 months			
					Change, mean (SD)			*P* value			Change, mean (SD)				*P* value
**Changes in the remote technology intervention group**															
	6MWT (m)			28.6 (44.3)			.01			32.9 (48.6)				.01	
	Body mass (kg)			−0.2 (2.8)			.78			−0.2 (3.4)				.75	
	BMI (kg/m^2^)			−0.03 (0.9)			.89			−0.1 (1.2)				.65	
	Waist circumference (cm)			−3.1 (2.6)			.01			−3 (3.1)				.01	
	**Qo** **L**														
		Physical domain (4-20)	0.4 (1.7)			.25			0.2 (1.6)				.57		
		Psychological domain (4-20)	0.2 (1.6)			.62			0.2 (1.8)				.53		
		Social relationships (4-20)	0.02 (2.2)			.96			−0.7 (2.1)				.11		
		Environmental domain (4-20)	0.5 (1.2)			.03			0.5 (1.4)				.11		
**Changes in the reference group**															
	6MWT (m)			20.3 (42.5)			.03			23.1 (40.5)				.01	
	Body mass (kg)			−0.2 (2.2)			.66			0.6 (2.5)				.27	
	BMI (kg/m^2^)			−0.07 (0.8)			.65			0.2 (0.8)				.24	
	Waist circumference (cm)			−1.5 (3)			.02			−0.8 (3.7)				.29	
	**Qo** **L**														
		Physical domain (4-20)	0 (2.1)			.95			−0.2 (2.9)				.71		
		Psychological domain (4-20)	−0.1 (1.3)			.69			0.3 (2.2)				.60		
		Social relationships (4-20)	0.5 (2.2)			.27			−0.4 (2.2)				.46		
		Environmental domain (4-20)	0.3 (1.2)			.33			0.2 (1.6)				.51		

## Discussion

### Principal Findings

In this study, cardiac rehabilitation including remote technology decreased waist circumference more than conventional rehabilitation without remote technology during the 6- and 12-month follow-ups. Our waist circumference results were similar to those reported in previous studies [29,30]. Widmer et al [29] found statistically significant reductions in waist circumference, body mass, and BMI. In contrast, we did not find statistically significant results for body mass or BMI, in accordance with the study by Dale et al [30]. The differences in intervention content may at least partly explain the contradictory results. Widmer et al [29] used self-monitoring through web- and smartphone-based cardiac rehabilitation platforms, whereas Dale et al [30] used automated daily text messages and access to a website.

Previous obesity-related, technology-based systematic reviews and meta-analyses in rehabilitation settings compared experimental groups with heterogeneous control groups [23]. The differences in the content of the experimental and control groups varied greatly, and because of this, the added value of the technology remains unclear. For example, in the study by Lahtio et al [23], none of the included studies examined the added value of technology. In most cases, for in-rehabilitation interventions, both technology and face-to-face meetings are used in experimental groups [23]. These were the main reasons why the interest in this study was to determine the added value of technology. Therefore, in this study, the only difference between the remote technology intervention and the reference groups was the use of technology. With this study’s design, it was possible to determine the added value of the technology.

Previous studies have demonstrated that a clinically significant change, 5% to 10% weight loss, is associated with health benefits [41], such as improvements in blood pressure [42,43], high density lipoprotein cholesterol [42], CVD risk factors [44]; and psychological changes, such as self-esteem and body satisfaction [45]. Therefore, sustained weight loss ranges from 3% to 5%, which can lead to clinically meaningful reductions in CVD risk factors [46]. It is important to note that both groups received rehabilitation and home exercises; however, the remote technology intervention group exhibited slightly better results than the reference group. The remote technology intervention and reference groups received small, positive, and statistically significant changes in waist circumference. However, there was only small nonsignificant changes in body mass: ‒0.2 kg (0.2%) in the remote technology intervention group and ‒0.2 kg (0.7%) in the reference group. However, these changes were not clinically significant.

However, in our study, statistically significant changes were observed in waist circumference, which is a clinically meaningful outcome in cardiac rehabilitation because increased waist circumference is associated with CVD risk and CVD mortality [36,47,48]. According to the Guidelines for Obesity Treatment (2015) [46], obesity treatment should focus not only on weight loss but also on waist circumference reduction and body composition improvement [41]. According to Ross et al [36], in each BMI category, participants with a high waist circumference had a greater risk of adverse health outcomes than those with normal waist circumference levels [36]. There are no general cutoff points for waist circumference [46]; however, in previous studies, a 5-cm reduction in waist circumference was found to be a clinically significant reduction [49], and a 10% waist circumference reduction was associated with improvements in blood pressure, lipids, and glycemia [42]. Correspondingly, every 5-cm increase in waist circumference increases the risk of death by 17% in men and 13% in women [36], and a 1-cm increase increases the future risk of CVDs by 2% [47]. Therefore, decreasing waist circumference is an important target for reducing adverse health risks because of its role in improving cardiometabolic risk factors [36]. In this study, the rehabilitees had a waist circumference reduction of 3 cm (3%) in the remote technology intervention group and 0.8 cm (0.8%) in the reference group at the 12-month time point. Therefore, according to our study, remote technology has the effect of decreasing waist circumference by 2.2 cm (9%). The findings in this study, namely, the changes in waist circumference, are not clinically relevant but support the idea that adding remote technology to cardiac rehabilitation may increase beneficial health outcomes.

The largest reduction in waist circumference occurred during the first 6 months in the remote technology intervention group. This result was also sustained at the 12-month measurement, but waist circumference did not decrease further during the 6- to 12-month period. These results are consistent with those of previous studies demonstrating that the largest weight reduction occurs during the first 6 months of the intervention [50,51]. However, it is important that the body mass, BMI, and waist circumference do not increase during rehabilitation. Preventing weight gain is a crucial behavioral strategy for achieving better health outcomes [52].

This study focused on weight loss more broadly than previous studies, including physical function and QoL. Only the environmental domain achieved a statistically significant between-group difference at the 6-month measurement point. Within-group differences in the 6MWT at every measurement point and in the environmental domain in the remote technology intervention group at the 6-month measurement point showed statistically significant changes. To our knowledge, only one study [53] has investigated weight loss, physical activity, and QoL in web-based weight loss programs. However, this study did not include cardiac rehabilitation and the outcome measurements differed from those in our study. The combination of weight loss, physical activity, and QoL is essential because physical activity can prevent CVDs, improve QoL, and help maintain a healthy body mass [14].

This study included social support from other rehabilitees and health care professionals twice a month. Rehabilitees also had their own Facebook groups; however, they were inactive in peer support, groups, and using Movendos. In this study, as the remote technology intervention and reference groups were randomized in the clusters, there was a possibility to evaluate whether there were statistically significant changes in the outcomes that occurred in the remote technology intervention (between clusters 2, 4, and 6) and reference (between clusters 1, 3, and 5) groups during the intervention (0-6 and 0-12 months). In this study, there were no statistically significant changes in any outcome between clusters. The sample size of the clusters was small, which could have affected the results. Therefore, the results of the cluster analyses were indicative. In the future, it is important to study the individual and group factors that affect the function of the group. A possible cluster effect should be considered in the future when planning and implementing rehabilitation interventions. For example, peer support has been found to be important in the process of weight loss, as rehabilitees learn together, share experiences, and feel a sense of belonging. They value the support of their peers in the same situation [54]. Social support is an important facilitator of behavioral change, and it raises the motivation and encouragement of rehabilitees [55].

### Strengths and Limitations

The strength of this study is that the conventional cardiac rehabilitation, rehabilitation staff, and background were the same in both the groups. Another strength is that this study evaluated the added value of technology in the everyday lives of rehabilitees in their home environments during self-rehabilitation periods. Most previous studies have evaluated the effectiveness of technology in clinical settings; however, in this study, we also investigated the effectiveness of rehabilitation during the self-rehabilitation period. A strength of this study is the comprehensive view it provides, in which the measurements were based on the biopsychosocial aspect of a rehabilitee. To the best of our knowledge, only one study [53] has investigated the association between physical activity, weight loss, and QoL in a web-based weight loss program in healthy adults. However, their study did not include cardiac rehabilitees.

A strength of this study is that cardiac rehabilitation was implemented by a multidisciplinary team. Previous studies have revealed that multidisciplinary rehabilitation is effective in body weight reduction [56-59]. The most effective weight loss management should include in-person, high-intensity comprehensive interventions that include individual and group sessions [46]. According to Blaž et al [59], a successful method for weight loss includes a multidisciplinary approach based on individual work with rehabilitees or groups. All these elements were included in this study. In technology-based weight loss rehabilitation, 5 components should be considered for achieving weight loss: self-monitoring, counselor feedback and communication, social support, structured programs, and individual programs [45,55]. Technology enables personalization by using different methods of weight loss interventions (eg, self-reporting, social support, and chat), which may enhance engagement with interventions [52]. The current service description of cardiac rehabilitation of the Social Insurance Institution of Finland is that cardiac rehabilitation enables peer and social support from health care professionals. Rehabilitation should be tailored according to the individual needs and goals of the rehabilitee [31].

The strength of this study was controlling for significant background factors of rehabilitation (similarity of rehabilitation context and seasonal effects) before intervention and the factors of the rehabilitees (comorbidities and cointervention) at baseline. There were only minor differences in the number of comorbidities and no statistically significant differences in the background factors of the rehabilitees at baseline. In addition, the rehabilitees did not show statistically significant lifestyle changes before the rehabilitation intervention. We used pairwise randomization in a consecutive order to control seasonal variety (winter, spring, and autumn), which may have caused systematic effect bias. Because we were able to control several confounding factors, the statistically significant changes in waist circumference and environmental domain may be associated with the added remote technology–assisted rehabilitation intervention.

A major limitation of this study is its small sample size, which affects its generalizability. However, the dropout rates were low in both groups. Because of the small sample size, we emphasized the guidance provided by the technology use. In this study, there was significant variance in the CIs. This could affect the internal validity of the study, which is typical of small studies. Another limitation of this study was blinding. It was not possible to blind the participants, caregivers, or outcome assessors because of the nature of the intervention. The protocol for cardiac rehabilitation was strictly standardized by the Social Insurance Institute of Finland, which is also responsible for rehabilitation costs. In addition, the risk of selection bias is present because the rehabilitees who participated in cardiac rehabilitation applied to the rehabilitation of their own will. In Finland, everyone who has undergone cardiac surgery has the opportunity to undergo rehabilitation. However, it is known that everyone who has undergone cardiac surgery does not apply for rehabilitation. One limitation is that we did not control for the use of health care services. The rehabilitees had normal health care services if needed.

According to the nature of the study protocol, the inclusion criteria were determined such that only severe functional ability problems were determined as exclusion criteria. The main purpose of the inclusion criteria was to ensure that the rehabilitees were capable of using technology regardless of the rehabilitation background. We kept the inclusion criteria broad and created an opportunity for a larger number of rehabilitees to participate in the study. All rehabilitees met the inclusion criteria. Real-life interdisciplinary rehabilitation study designs are valuable for producing new information on rehabilitation that can be implemented directly in real-life situations and environments, compared with laboratory research. Unfortunately, in a real environment, we must make concessions in some aspects of study design, such as determining the sample size of the study population and using cluster randomization instead of randomization at individual level. In the future, with a larger study group, it would be important to study more specifically the effects of individual factors, such as medication or comorbidities, in added value of technology-based rehabilitation.

Because there was some level of systematic (blinding and selection bias) error during the intervention and samples were relatively small, care must be taken in generalizing the study results beyond the target population. In order to confirm the perceptions of the added value of remote technology in rehabilitation interventions, more studies involving rehabilitees with cardiac rehabilitation are required.

Several measurements were used in this cluster randomized trial, which are widely used, but have some limitations. In the 6MWT, small changes in the test methodology can affect the results. Therefore, the measurer should carefully follow the test protocol [32]. The 6MWT can provide reliable information about the daily activities of a rehabilitee [33], and with it, the submaximal level of functional capacity can be assessed. However, it does not test peak oxygen uptake and cannot replace the maximal exercise test [60]. BMI alone is an imprecise measurement, as it is an inadequate marker of abdominal fat [36] and does not separate muscle mass weight from fat mass [61]. The limitations of waist circumference are its inaccuracy in separating subcutaneous fat from visceral fat [62] and its cutoff points, which cannot be generalized universally [63]. Waist circumference has been used with BMI to evaluate overweight and obesity [36]. It is widely used because it is associated with all-cause and cardiovascular mortality [36]. Waist circumference is a simple measurement that is an appropriate index of intra-abdominal fat mass [61]. The WHOQOL-BREF is a valid and reliable measurement and has been studied in a wide variety of nations and diseases. However, some previous studies have reported a ceiling effect in the WHOQOL-BREF, which means that items only measure negative changes, not positive changes [64,65]. One limitation is that the social domain has only a few items [64] and may have low reliability [65].

Although weight loss has been widely studied, we still do not understand all the aspects of weight loss. Therefore, in the future, it would be important to study the factors considered essential for cardiac or obese rehabilitation to help maintain motivation for weight loss. Social support and the amount of technology used are relevant to the effectiveness of an intervention [55,66]. Therefore, these factors should be investigated further in future studies. It is important to identify the factors that increase the motivation for rehabilitation after 6 months, which is when the greatest weight loss typically occurs. The role of remote technology in cardiac rehabilitation is important to study further, and the question of whether remote technology aids in maintaining motivation should also be further investigated. It is also important to study the long-term effects of cardiac rehabilitation with technology on body composition and to study the effects that remain after the intervention and perform follow-up measurements. In addition, it would be of interest to determine whether the rehabilitees use technological equipment, what the effects of the equipment are, and whether they have managed to achieve their weight loss goal.

### Conclusions

This study demonstrated that remote cardiac rehabilitation added value to conventional cardiac rehabilitation in terms of waist circumference and environmental QoL; however, the results were clinically relatively small. There were no statistically significant differences between the remote technology intervention and reference groups in the 6MWT; body mass; BMI; and physical, psychological, and social domains in the WHOQOL-BREF. These findings support the idea that adding remote technology to cardiac rehabilitation may improve beneficial health outcomes. There was some level of systematic (blinding and selection bias) error during the rehabilitation intervention and samples were relatively small; care must be taken in generalizing the study results beyond the target population. To confirm the assumptions of the added value of remote technology in rehabilitation interventions, more randomized and controlled follow-up studies involving rehabilitees with different cardiac rehabilitation are required.

## Appendix

Multimedia Appendix 1 Consort eHealth V1.6.

## Abbreviations

CVD: cardiovascular disease
QoL: quality of life
WHOQOL-BREF: World Health Organization quality of life-BREF
6MWT: 6-minute walk test

## Data Availability

All data generated or analyzed during this study are included in this published article.

## References

[1] Hakala S, Rintala A, Immonen J, Karvanen J, Heinonen A, Sjögren T (2017). Effectiveness of physical activity promoting technology-based distance interventions compared to usual care. Systematic review, meta-analysis and meta-regression. Eur J Phys Rehabil Med.

[2] Free C, Phillips G, Galli L, Watson L, Felix L, Edwards P, Patel V, Haines A (2013). The effectiveness of mobile-health technology-based health behaviour change or disease management interventions for health care consumers: a systematic review. PLoS Med.

[3] (2021). Cardiovascular diseases (CVDs). World Health Organization.

[4] (2020). WHO reveals leading causes of death and disability worldwide: 2000-2019. World Health Organization.

[5] Salminen AL (2022). Omakuntoutuksesta yksilön hyvinvoinnin hallintaan. Helsinki: Social Insurance Institution of Finland.

[6] Huang K, Liu W, He D, Huang B, Xiao D, Peng Y, He Y, Hu H, Chen M, Huang D (2015). Telehealth interventions versus center-based cardiac rehabilitation of coronary artery disease: a systematic review and meta-analysis. Eur J Prev Cardiol.

[7] Varnfield M, Karunanithi M, Lee CK, Honeyman E, Arnold D, Ding H, Smith C, Walters DL (2014). Smartphone-based home care model improved use of cardiac rehabilitation in postmyocardial infarction patients: results from a randomised controlled trial. Heart.

[8] Roth GA, Mensah GA, Johnson CO, Addolorato G, Ammirati E, Baddour LM, Barengo NC, Beaton AZ, Benjamin EJ, Benziger CP, Bonny A, Brauer M, Brodmann M, Cahill TJ, Carapetis J, Catapano AL, Chugh SS, Cooper LT, Coresh J, Criqui M, DeCleene N, Eagle KA, Emmons-Bell S, Feigin VL, Fernández-Solà J, Fowkes G, Gakidou E, Grundy SM, He FJ, Howard G, Hu F, Inker L, Karthikeyan G, Kassebaum N, Koroshetz W, Lavie C, Lloyd-Jones D, Lu HS, Mirijello A, Temesgen AM, Mokdad A, Moran AE, Muntner P, Narula J, Neal B, Ntsekhe M, Moraes de Oliveira G, Otto C, Owolabi M, Pratt M, Rajagopalan S, Reitsma M, Ribeiro AL, Rigotti N, Rodgers A, Sable C, Shakil S, Sliwa-Hahnle K, Stark B, Sundström J, Timpel P, Tleyjeh IM, Valgimigli M, Vos T, Whelton PK, Yacoub M, Zuhlke L, Murray C, Fuster V, GBD-NHLBI-JACC Global Burden of Cardiovascular Diseases Writing Group (2020). Global burden of cardiovascular diseases and risk factors, 1990-2019: update from the GBD 2019 study. J Am Coll Cardiol.

[9] (2010). Body mass index. World Health Organization.

[10] Csige I, Ujvárosy D, Szabó Z, Lőrincz I, Paragh G, Harangi M, Somodi S (2018). The impact of obesity on the cardiovascular system. J Diabetes Res.

[11] Koliaki C, Liatis S, Kokkinos A (2019). Obesity and cardiovascular disease: revisiting an old relationship. Metabolism.

[12] Du H, Newton PJ, Salamonson Y, Carrieri-Kohlman VL, Davidson PM (2009). A review of the six-minute walk test: its implication as a self-administered assessment tool. Eur J Cardiovasc Nurs.

[13] Mehra VM, Gaalema DE, Pakosh M, Grace SL (2020). Systematic review of cardiac rehabilitation guidelines: quality and scope. Eur J Prev Cardiol.

[14] (2022). Physical activity. World Health Organization.

[15] Coronary heart disease. Käypä hoito.

[16] Thomas RJ, Beatty AL, Beckie TM, Brewer LC, Brown TM, Forman DE, Franklin BA, Keteyian SJ, Kitzman DW, Regensteiner JG, Sanderson BK, Whooley MA (2019). Home-based cardiac rehabilitation: a scientific statement from the American association of cardiovascular and pulmonary rehabilitation, the American Heart Association, and the American College of Cardiology. J Am Coll Cardiol.

[17] Imran HM, Baig M, Erqou S, Taveira TH, Shah NR, Morrison A, Choudhary G, Wu WC (2019). Home-based cardiac rehabilitation alone and hybrid with center-based cardiac rehabilitation in heart failure: a systematic review and meta-analysis. J Am Heart Assoc.

[18] Salminen AL, Hiekkala S, Stenberg JH (2016). Etäkuntoutus. Kelan Tutkimus.

[19] Hakala S, Rintala A, Immonen J, Karvanen J, Heinonen A, Sjögren T (2017). Effectiveness of technology-based distance interventions promoting physical activity: systematic review, meta-analysis and meta-regression. J Rehabil Med.

[20] Buckingham SA, Taylor RS, Jolly K, Zawada A, Dean SG, Cowie A, Norton RJ, Dalal HM (2016). Home-based versus centre-based cardiac rehabilitation: abridged Cochrane systematic review and meta-analysis. Open Heart.

[21] Anderson L, Sharp GA, Norton RJ, Dalal H, Dean SG, Jolly K, Cowie A, Zawada A, Taylor RS (2017). Home-based versus centre-based cardiac rehabilitation. Cochrane Database Syst Rev.

[22] Hakala S, Kivistö H, Paajanen T, Kankainen A, Anttila MR, Heinonen A, Sjögren T (2021). Effectiveness of distance technology in promoting physical activity in cardiovascular disease rehabilitation: cluster randomized controlled trial, a pilot study. JMIR Rehabil Assist Technol.

[23] Lahtio H, Rintala A, Immonen J, Sjögren T (2022). The effectiveness of physical activity-promoting web- and mobile-based distance weight loss interventions on body composition in rehabilitation settings: systematic review, meta-analysis, and meta-regression analysis. J Med Internet Res.

[24] Piette JD, List J, Rana GK, Townsend W, Striplin D, Heisler M (2015). Mobile health devices as tools for worldwide cardiovascular risk reduction and disease management. Circulation.

[25] Dorje T, Zhao G, Tso K, Wang J, Chen Y, Tsokey L, Tan BK, Scheer A, Jacques A, Li Z, Wang R, Chow CK, Ge J, Maiorana A (2019). Smartphone and social media-based cardiac rehabilitation and secondary prevention in China (SMART-CR/SP): a parallel-group, single-blind, randomised controlled trial. Lancet Digit Health.

[26] Halldorsdottir H, Thoroddsen A, Ingadottir B (2020). Impact of technology-based patient education on modifiable cardiovascular risk factors of people with coronary heart disease: a systematic review. Patient Educ Couns.

[27] Imran TF, Wang N, Zombeck S, Balady GJ (2021). Mobile technology improves adherence to cardiac rehabilitation: a propensity score-matched study. J Am Heart Assoc.

[28] Lounsbury P, Elokda AS, Gylten D, Arena R, Clarke W, Gordon EE (2015). Text-messaging program improves outcomes in outpatient cardiovascular rehabilitation. Int J Cardiol Heart Vasc.

[29] Widmer RJ, Allison TG, Lennon R, Lopez-Jimenez F, Lerman LO, Lerman A (2017). Digital health intervention during cardiac rehabilitation: a randomized controlled trial. Am Heart J.

[30] Pfaeffli Dale L, Whittaker R, Jiang Y, Stewart R, Rolleston A, Maddison R (2015). Text message and internet support for coronary heart disease self-management: results from the Text4Heart randomized controlled trial. J Med Internet Res.

[31] (2021). Aikuisen sydänkuntoutuskurssi. Kela.

[32] Holland AE, Spruit MA, Troosters T, Puhan MA, Pepin V, Saey D, McCormack MC, Carlin BW, Sciurba FC, Pitta F, Wanger J, MacIntyre N, Kaminsky DA, Culver BH, Revill SM, Hernandes NA, Andrianopoulos V, Camillo CA, Mitchell KE, Lee AL, Hill CJ, Singh SJ (2014). An official European respiratory society/American thoracic society technical standard: field walking tests in chronic respiratory disease. Eur Respir J.

[33] Giannitsi S, Bougiakli M, Bechlioulis A, Kotsia A, Michalis LK, Naka KK (2019). 6-minute walking test: a useful tool in the management of heart failure patients. Ther Adv Cardiovasc Dis.

[34] Du H, Wonggom P, Tongpeth J, Clark RA (2017). Six-minute walk test for assessing physical functional capacity in chronic heart failure. Curr Heart Fail Rep.

[35] (2021). Obesity and overweight. World Health Organization.

[36] Ross R, Neeland IJ, Yamashita S, Shai I, Seidell J, Magni P, Santos RD, Arsenault B, Cuevas A, Hu FB, Griffin BA, Zambon A, Barter P, Fruchart JC, Eckel RH, Matsuzawa Y, Després JP (2020). Waist circumference as a vital sign in clinical practice: a consensus statement from the IAS and ICCR working group on visceral obesity. Nat Rev Endocrinol.

[37] Body mass index and waist circumference. Finnish Medical Society Duodecim.

[38] Ades PA, Savage PD (2017). Obesity in coronary heart disease: an unaddressed behavioral risk factor. Prev Med.

[39] Ko HY, Lee JK, Shin JY, Jo E (2015). Health-related quality of life and cardiovascular disease risk in Korean adults. Korean J Fam Med.

[40] WHOQOL: measuring quality of life. World Health Organization.

[41] Yumuk V, Tsigos C, Fried M, Schindler K, Busetto L, Micic D, Toplak H, Obesity Management Task Force of the European Association for the Study of Obesity (2015). European guidelines for obesity management in adults. Obes Facts.

[42] Rothberg AE, McEwen LN, Kraftson AT, Ajluni N, Fowler CE, Nay CK, Miller NM, Burant CF, Herman WH (2017). Impact of weight loss on waist circumference and the components of the metabolic syndrome. BMJ Open Diabetes Res Care.

[43] Ryan DH, Yockey SR (2017). Weight loss and improvement in comorbidity: differences at 5%, 10%, 15%, and over. Curr Obes Rep.

[44] Wing RR, Lang W, Wadden TA, Safford M, Knowler WC, Bertoni AG, Hill JO, Brancati FL, Peters A, Wagenknecht L, Look AHEAD Research Group (2011). Benefits of modest weight loss in improving cardiovascular risk factors in overweight and obese individuals with type 2 diabetes. Diabetes Care.

[45] Castelnuovo G, Manzoni GM, Pietrabissa G, Corti S, Giusti EM, Molinari E, Simpson S (2014). Obesity and outpatient rehabilitation using mobile technologies: the potential mHealth approach. Front Psychol.

[46] American College of Cardiology/American Heart Association Task Force on Practice Guidelines‚ Obesity Expert Panel‚ 2013 (2014). Executive summary: guidelines (2013) for the management of overweight and obesity in adults: a report of the American College of Cardiology/American Heart Association Task Force on Practice Guidelines and the Obesity Society published by the Obesity Society and American College of Cardiology/American Heart Association Task Force on practice guidelines. Based on a systematic review from the The Obesity Expert Panel, 2013. Obesity (Silver Spring).

[47] Mulligan AA, Lentjes MA, Luben RN, Wareham NJ, Khaw KT (2019). Changes in waist circumference and risk of all-cause and CVD mortality: results from the European Prospective Investigation into Cancer in Norfolk (EPIC-Norfolk) cohort study. BMC Cardiovasc Disord.

[48] Czernichow S, Kengne AP, Stamatakis E, Hamer M, Batty GD (2011). Body mass index, waist circumference and waist-hip ratio: which is the better discriminator of cardiovascular disease mortality risk?: evidence from an individual-participant meta-analysis of 82 864 participants from nine cohort studies. Obes Rev.

[49] Verweij LM, Terwee CB, Proper KI, Hulshof CT, van Mechelen W (2013). Measurement error of waist circumference: gaps in knowledge. Public Health Nutr.

[50] Jakicic JM, Davis KK, Rogers RJ, King WC, Marcus MD, Helsel D, Rickman AD, Wahed AS, Belle SH (2016). Effect of wearable technology combined with a lifestyle intervention on long-term weight loss: the IDEA randomized clinical trial. JAMA.

[51] Franz MJ, VanWormer JJ, Crain AL, Boucher JL, Histon T, Caplan W, Bowman JD, Pronk NP (2007). Weight-loss outcomes: a systematic review and meta-analysis of weight-loss clinical trials with a minimum 1-year follow-up. J Am Diet Assoc.

[52] Laranjo L, Quiroz JC, Tong HL, Arevalo Bazalar M, Coiera E (2020). A mobile social networking app for weight management and physical activity promotion: results from an experimental mixed methods study. J Med Internet Res.

[53] Höchsmann C, Dorling JL, Martin CK, Earnest CP, Church TS (2022). Association between weight loss, change in physical activity, and change in quality of life following a corporately sponsored, online weight loss program. BMC Public Health.

[54] Anttila MR, Söderlund A, Sjögren T (2021). Patients' experiences of the complex trust-building process within digital cardiac rehabilitation. PLoS One.

[55] Khaylis A, Yiaslas T, Bergstrom J, Gore-Felton C (2010). A review of efficacious technology-based weight-loss interventions: five key components. Telemed J E Health.

[56] Lu D, Yuan Z, Yang L, Jiang Y, Li M, Wang Y, Jing L, Wang R, Zhang J, Guo X (2021). Body composition and metabolic improvement in patients followed up by a multidisciplinary team for obesity in China. J Diabetes Res.

[57] Foster D, Sanchez-Collins S, Cheskin LJ (2017). Multidisciplinary team-based obesity treatment in patients with diabetes: current practices and the state of the science. Diabetes Spectr.

[58] Candemir I, Ergun P, Kaymaz D (2017). Efficacy of a multidisciplinary pulmonary rehabilitation outpatient program on exacerbations in overweight and obese patients with asthma. Wien Klin Wochenschr.

[59] Kovač Blaž M, Švab I (2015). A multidisciplinary approach to treating obesity in a community health centre. Zdr Varst.

[60] ATS Committee on Proficiency Standards for Clinical Pulmonary Function Laboratories (2002). ATS statement: guidelines for the six-minute walk test. Am J Respir Crit Care Med.

[61] (2000). Obesity: preventing and managing the global epidemic: report of a WHO consultation. World Health Organization.

[62] Fox CS, Massaro JM, Hoffmann U, Pou KM, Maurovich-Horvat P, Liu CY, Vasan RS, Murabito JM, Meigs JB, Cupples LA, D'Agostino RB, O'Donnell CJ (2007). Abdominal visceral and subcutaneous adipose tissue compartments: association with metabolic risk factors in the Framingham heart study. Circulation.

[63] (2008). Waist circumference and waist-hip ratio: report of a WHO expert consultation. World Health Organization.

[64] Rosén H, Ahlström G, Lexén A (2020). Psychometric properties of the WHOQOL-BREF among next of kin to older persons in nursing homes. Health Qual Life Outcomes.

[65] Kalfoss MH, Reidunsdatter RJ, Klöckner CA, Nilsen M (2021). Validation of the WHOQOL-Bref: psychometric properties and normative data for the Norwegian general population. Health Qual Life Outcomes.

[66] Haapala I, Barengo NC, Biggs S, Surakka L, Manninen P (2009). Weight loss by mobile phone: a 1-year effectiveness study. Public Health Nutr.

