# Supplementary material for: The Added Value of Remote Technology in Cardiac Rehabilitation on Physical Function, Anthropometrics, and Quality of Life: Cluster Randomized Controlled Trial
Source: J Med Internet Res. 2023 Apr 12;25:e42455. doi: 10.2196/42455 (PMC10134015; doi:10.2196/42455)
Supplement: Multimedia Appendix 1 [file jmir_v25i1e42455_app1.pdf]

CONSORT-EHEALTH Checklist V1.6.2 Report	Manuscript Number	42455
**(based on CONSORT-EHEALTH V1.6), available at [http://tinyurl.com/consort-ehealth-v1-6].**		
**Date completed**		
4/5/2023 16:10:27		
**by**		
Heli Lahtio		
The Added Value of Remote Technology in Cardiac Rehabilitation on Physical Function, Anthropometrics, and Quality of Life: Cluster Randomized Controlled Trial		
TITLE		
**1a-i) Identify the mode of delivery in the title**		
We used "The Added Value of Remote Technology" because we had an app and an activity tracker and we wanted to examine the added value of remote technology		
**1a-ii) Non-web-based components or important co-interventions in title**		
**1a-iii) Primary condition or target group in the title**		
We used "Cardiac rehabilitation" in our title		
ABSTRACT		
**1b-i) Key features/functionalities/components of the intervention and comparator in the METHODS section of the ABSTRACT**		
"The reference group received conventional cardiac rehabilitation, and the remote technology intervention group received conventional cardiac rehabilitation with added remote technology, namely, the Movendos m-coach-app and Fitbit Charge accelerometer. The 12 months of rehabilitation consisted of three 5-day in-rehabilitation periods in the rehabilitation center. Between these periods were two 6-month self-rehabilitation periods. Outcome measurements included the 6-minute walk test, body mass, BMI, waist circumference, and the WHOQOL-BREF questionnaire at baseline and 6 months and 12 months. "		
**1b-ii) Level of human involvement in the METHODS section of the ABSTRACT**		
The rehabilitees received cardiac rehabilitation which included appointments with several healthcare professionals. In addition, the intervention group received technology which they used independently. Therefore, we did not mention it in the abstract.		
**1b-iii) Open vs. closed, web-based (self-assessment) vs. face-to-face assessments in the METHODS section of the ABSTRACT**		
In the abstract, there is a word limit. Therefore, it is not possible to describe the recruitment process in the abstract. However, we have described that "The 12 months of rehabilitation consisted of three 5-day in-rehabilitation periods in the rehabilitation center. Between these periods were two 6-month self-rehabilitation periods." We used WHOQOL-BREF questionnaire as an outcome measurement. However, the participants fulfilled the questionnaires in the rehabilitation center. Therefore, we do not want to use a term "self-reported".		
**1b-iv) RESULTS section in abstract must contain use data**		
The number of participants and results has been described: "Overall, 59 rehabilitees aged 41 to 66 years (mean age 60, SD 6 years; n=48, 81% men) were included in the study. Decrement in waist circumference (6 months: 1.6 cm, P=.04; 12 months: 3 cm, P<.001) and increment in self-assessed QoL were more (environmental factors, 0.5; P=.02) in the remote technology intervention group than those in the reference group. Both groups achieved statistically significant improvements in the 6-minute walk test in both time frames. In addition, the remote technology intervention group achieved statistically significant changes in environmental domain at 0-6 months and in waist circumference at both time frames, and reference group in 0-6 months."		
**1b-v) CONCLUSIONS/DISCUSSION in abstract for negative trials**		
There was statistically significant changes in waist circumference and QoL so the intervention was not negative.		
INTRODUCTION		
**2a-i) Problem and the type of system/solution**		
The problem, patient population and goals have been described in the introduction. "This study aimed to investigate at the individual level the added value of remote technology in cardiac rehabilitation on physical function, anthropometrics, and QoL in participants with CVD compared with conventional rehabilitation. Rehabilitees attended standard group-based cardiac rehabilitation courses at a rehabilitation center in Finland."		
**2a-ii) Scientific background, rationale: What is known about the (type of) system**		
Scientific background has been described. We have discussed of CVD, cardiac rehabilitation, and technology in rehabilitation which are the main themes in our study.		
**Does your paper address CONSORT subitem 2b?**		
Objective has been described in our study: "This study aimed to investigate at the individual level the added value of remote technology in cardiac rehabilitation on physical function, anthropometrics, and QoL in participants with CVD compared with conventional rehabilitation."		
METHODS		
**3a) CONSORT: Description of trial design (such as parallel, factorial) including allocation ratio**		
Trial design has been described: "This study was a cluster randomized trial. Recruitment and data collection were conducted between September 2015 and May 2017 at the Finnish Rehabilitation Center. This study was a real-life research project with 1-year data recruitment period.<br>Rehabilitees were distributed into 6 groups by officers of the Social Insurance Institution of Finland. Randomization occurred at the group level in pairs of 2 consecutive groups with following two experimental arms: (1) conventional cardiac rehabilitation with remote technology (remote technology intervention) group and (2) conventional cardiac rehabilitation (reference) group. The rehabilitees were randomized into 3 remote technology intervention clusters and 3 reference clusters."		
**3b) CONSORT: Important changes to methods after trial commencement (such as eligibility criteria), with reasons**		
Eligibility criteria were determined before the trial commencement. Therefore, changes to them did not occur. Eligibility criteria has been described in methods.		
**3b-i) Bug fixes, Downtimes, Content Changes**		
This study was arranged by Social Insurance Institution of Finland and it lasted 12 months. No bug fixes, downtimes or content changes occurred during the intervention.		
**4a) CONSORT: Eligibility criteria for participants**		
Eligibility criteria for participants has been described: "The primary eligibility criterion for the study was an adult (aged <18 years) with coronary heart disease and having independent basic level of management of IT and remote technology applications."		
**4a-i) Computer / Internet literacy**		
Eligibility criteria was "basic level of management of IT and remote technology applications".		
**4a-ii) Open vs. closed, web-based vs. face-to-face assessments:**		
"The rehabilitees attended to coronary heart disease rehabilitation courses in rehabilitation center and they applied for the course with a physician's referral". The intervention content has been explained in Methods. Technological equipments were Movendos m-coach app and Fitbit Charge HR accelerometer. The rehabilitation also included in-rehabilitation periods when the rehabilitees were in the rehabilitation center. Rehabilitation included e.g. meetings with physiotherapist, social worker, physician and psychologist. The researchers were in contact with the rehabilitees by phone. "Because of the nature of the intervention, it was not possible to blind the rehabilitees and caregivers in terms of the intervention. The outcome assessor was not blinded to the intervention, but one educated person who was not involved in the study group performed the anthropometric measurements, and another person performed the 6-minute walk tests (6MWTs). The statistician was blinded to the interventions."		
**4a-iii) Information giving during recruitment**		
The rehabilitees attended to cardiac rehabilitation with physician's referral.		
**4b) CONSORT: Settings and locations where the data were collected**		
Settings and locations have been described in methods: "Measurements were performed at the rehabilitation center at baseline and at the 6- and 12-month measurement points. Anthropometrics were measured by a nurse, 6MWT by physiotherapists, and WHOQOL-BREF questionnaires by the researchers."		
**4b-i) Report if outcomes were (self-)assessed through online questionnaires**		
WHOQOL-BREF questionnaire was self filled by the rehabilitees but they filled it in the rehabilitation center with the guidance of the researchers.		
**4b-ii) Report how institutional affiliations are displayed**		
**5) CONSORT: Describe the interventions for each group with sufficient details to allow replication, including how and when they were actually administered**		
**5-i) Mention names, credential, affiliations of the developers, sponsors, and owners**		
There were no conflict of interests.		
**5-ii) Describe the history/development process**		
There was not any development process of an application.		
**5-iii) Revisions and updating**		
There was not any development process of an application or an intervention.		
**5-iv) Quality assurance methods**		
There was not any development process of an application or an intervention.		
**5-v) Ensure replicability by publishing the source code, and/or providing screenshots/screen-capture video, and/or providing flowcharts of the algorithms used**		

There was not any development process of an application or an intervention.		
**5-vi) Digital preservation**		
There was not any development process of an application or an intervention.		
**5-vii) Access**		
There was not any development process of an application or an intervention.		
**5-viii) Mode of delivery, features/functionalities/components of the intervention and comparator, and the theoretical framework**		
There was not any development process of an application or an intervention.		
**5-ix) Describe use parameters**		
"The accelerometers were guided to worn daily."		
**5-x) Clarify the level of human involvement**		
The rehabilitees attended to cardiac rehabilitation which included meetings with healthcare professionals. The researchers contacted the rehabilitees during the self-rehabilitation periods.		
**5-xi) Report any prompts/reminders used**		
Movendos sent automatic motivational reminders monthly.		
**5-xii) Describe any co-interventions (incl. training/support)**		
This study was not stand-alone intervention and there were no co-interventions. "In Finland, it is possible to participate in only 1 rehabilitation intervention at the same time as the Social Insurance Institute of Finland affirmed rehabilitation interventions." "Rehabilitees received information and pamphlets regarding CVDs and the management of daily activities, such as dietary habits, relaxation, physical activity, social security benefits, self-care, and self-rehabilitation while living with CVDs."		
**6a) CONSORT: Completely defined pre-specified primary and secondary outcome measures, including how and when they were assessed**		
"This secondary study used individually measured outcomes, which were chosen based on the following biopsychosocial model: the 6MWT, BMI, waist circumference, and the QoL-BREF (World Health Organization QoL-BREF [WHOQOL-BREF]) questionnaire. Measurements were performed at the rehabilitation center at baseline and at the 6- and 12-month measurement points. Anthropometrics were measured by a nurse, 6MWT by physiotherapists, and WHOQOL-BREF questionnaires by the researchers."		
**6a-i) Online questionnaires: describe if they were validated for online use and apply CHERRIES items to describe how the questionnaires were designed/deployed**		
There were no online questionnaires.		
**6a-ii) Describe whether and how "use" (including intensity of use/dosage) was defined/measured/monitored**		
Use was not measured during this intervention.		
**6a-iii) Describe whether, how, and when qualitative feedback from participants was obtained**		
Feedback from the rehabilitees was obtained in focus group interviews but they are not included in this article.		
**6b) CONSORT: Any changes to trial outcomes after the trial commenced, with reasons**		
Settings and locations have been described in methods: "Measurements were performed at the rehabilitation center at baseline and at the 6- and 12-month measurement points. Anthropometrics were measured by a nurse, 6MWT by physiotherapists, and WHOQOL-BREF questionnaires by the researchers."		
**7a) CONSORT: How sample size was determined**		
**7a-i) Describe whether and how expected attrition was taken into account when calculating the sample size**		
Sample size was not calculated because this was a real-life study where we included all the rehabilitees that possible. Social Insurance Institute of Finland recruited all rehabilitees. We have discussed this in discussion. "Unfortunately, in a real environment, we must make concessions in some aspects of study design, such as determining the sample size"		
**7b) CONSORT: When applicable, explanation of any interim analyses and stopping guidelines**		
"This secondary study used individually measured outcomes, which were chosen based on the following biopsychosocial model: the 6MWT, BMI, waist circumference, and the QoL-BREF (World Health Organization QoL-BREF [WHOQOL-BREF]) questionnaire. Measurements were performed at the rehabilitation center at baseline and at the 6- and 12-month measurement points. Anthropometrics were measured by a nurse, 6MWT by physiotherapists, and WHOQOL-BREF questionnaires by the researchers."		
**8a) CONSORT: Method used to generate the random allocation sequence**		
The same healthcare providers and researcher were responsible for every trial group. There were no differences in different groups.		
**8b) CONSORT: Type of randomisation; details of any restriction (such as blocking and block size)**		
"Randomization was accomplished with sealed envelopes within rehabilitation groups, and a person outside the research group (researcher of the gerontological research center) processed the randomization under the supervision of 2 researchers (TS and Heikki Kivistö). Randomization was performed 3 times for 2 consecutive groups, considering the season and months. In total, 59 rehabilitees were randomly assigned in pairs into (1) the remote technology intervention group (n=29) or (2) the reference group (n=30) in consecutive order."		
**9) CONSORT: Mechanism used to implement the random allocation sequence (such as sequentially numbered containers), describing any steps taken to conceal the sequence until interventions were assigned**		
"Randomization was accomplished with sealed envelopes within rehabilitation groups, and a person outside the research group (researcher of the gerontological research center) processed the randomization under the supervision of 2 researchers (TS and Heikki Kivistö). Randomization was performed 3 times for 2 consecutive groups, considering the season and months. In total, 59 rehabilitees were randomly assigned in pairs into (1) the remote technology intervention group (n=29) or (2) the reference group (n=30) in consecutive order."		
**10) CONSORT: Who generated the random allocation sequence, who enrolled participants, and who assigned participants to interventions**		
A person outside the study performed the randomization. All participants fulfilled eligibility criteria. Therefore, every rehabilitee were able to join to the study. "At the beginning of the rehabilitation, the researchers (TS and HK) informed the rehabilitees about the group (n=6) their rehabilitation group had been randomized."		
**11a) CONSORT: Blinding - If done, who was blinded after assignment to interventions (for example, participants, care providers, those assessing outcomes) and how**		
**11a-i) Specify who was blinded, and who wasn't**		
"Because of the nature of the intervention, it was not possible to blind the rehabilitees and caregivers in terms of the intervention. The outcome assessor was not blinded to the intervention, but one educated person who was not involved in the study group performed the anthropometric measurements, and another person performed the 6-minute walk tests (6MWTs). The statistician was blinded to the interventions."		
**11a-ii) Discuss e.g., whether participants knew which intervention was the "intervention of interest" and which one was the "comparator"**		
Both groups received the same intervention with the exception that the intervention group received technology and the control group did not. Therefore, the rehabilitees knew that were they in the intervention or control group. We have discussed this in the discussion: "Because there was some level of systematic (blinding, selection bias) error during the intervention and samples were relatively small, care must be taken in generalizing the study results beyond the target population."		
**11b) CONSORT: If relevant, description of the similarity of interventions**		
Both groups received the same intervention with the exception that the intervention group received technology and the control group did not. With this study design it was possible to study the effectiveness of technology.		
**12a) CONSORT: Statistical methods used to compare groups for primary and secondary outcomes**		
"Between-group differences were assessed using 2-tailed t tests and Mann-Whitney U tests. The normality of the groups had to be tested because the sample size in both groups was <50. Normality was tested using the Shapiro-Wilk test. If the P value was >.05, an independent samples t test was performed; otherwise, the Mann-Whitney U test was used. Variance in group equality was tested using Levene test. This was used in the case of the independent samples t test. If the P value was <.05 Levene test, 1-way ANOVA was performed. Otherwise, the variances were assumed to be equal and 2-way ANOVA was performed. Changes within groups were analyzed using a paired samples t test or Wilcoxon signed-rank test. In addition, between-clusters (1-6) differences were tested using 1-way ANOVA and Tukey test (0-6 and 0-12 months)."		
**12a-i) Imputation techniques to deal with attrition / missing values**		
Imputation was not performed due to small sample size. Analyses included only the results of the rehabilitees.		
**12b) CONSORT: Methods for additional analyses, such as subgroup analyses and adjusted analyses**		
"Changes within groups were analyzed using a paired samples t test or Wilcoxon signed-rank test. In addition, between-clusters (1-6) differences were tested using 1-way ANOVA and Tukey test (0-6 and 0-12 months)."		
RESULTS		
**13a) CONSORT: For each group, the numbers of participants who were randomly assigned, received intended treatment, and were analysed for the primary outcome**		
A flowchart has been introduced in the article. Only 6 rehabilitees discontinued the intervention. "Among the 59 rehabilitees, the dropout rate was 10% over 12 months period (Figure 2). At the 6-month follow-up point, 2 and 3 rehabilitees dropped out of the remote technology intervention and reference groups, respectively. At the 12-month follow-up, only 1 rehabilitee from the remote technology intervention group withdrew from the study."		
**13b) CONSORT: For each group, losses and exclusions after randomisation, together with reasons**		
The dropouts and reasons has been presented in Flowchart (Figure 2).		
**13b-i) Attrition diagram**		
An attrition rate was not essential in this study. The rehabilitees used activity trackers daily, and they had monthly tasks to perform. "The app was used in home exercise instructions, advice, and monitoring of the rehabilitees."		
**14a) CONSORT: Dates defining the periods of recruitment and follow-up**		
"The clusters began in autumn (September to November), winter (December to February), and spring (March to May)." The outcome assessment was performed at baseline, at 6-months and at 12-months.		

**14a-i) Indicate if critical “secular events” fell into the study period**		
"No serious adverse events occurred during the study."		
**14b) CONSORT: Why the trial ended or was stopped (early)**		
Trial was not ended early.		
**15) CONSORT: A table showing baseline demographic and clinical characteristics for each group**		
Table 1 shows the demographic and clinical characteristics of intervention and control groups.		
**15-i) Report demographics associated with digital divide issues**		
Age, education, gender, and social-economic status has been described in table 1. The basic level of management of IT was an eligibility criteria.		
**16a) CONSORT: For each group, number of participants (denominator) included in each analysis and whether the analysis was by original assigned groups**		
**16-i) Report multiple “denominators” and provide definitions**		
The number of participants has been described in table 2. This study proceeded with a clear structure and every rehabilitee followed the structure.		
**16-ii) Primary analysis should be intent-to-treat**		
Analysis was intent-to-treat analysis.		
**17a) CONSORT: For each primary and secondary outcome, results for each group, and the estimated effect size and its precision (such as 95% confidence interval)**		
Results, SDs and 95% confidence intervals have been presented in table 2.		
**17a-i) Presentation of process outcomes such as metrics of use and intensity of use**		
This is not relevant in our study.		
**17b) CONSORT: For binary outcomes, presentation of both absolute and relative effect sizes is recommended**		
This is not relevant in our study.		
**18) CONSORT: Results of any other analyses performed, including subgroup analyses and adjusted analyses, distinguishing pre-specified from exploratory**		
Within-group changes and between-clusters analyses were performed.		
**18-i) Subgroup analysis of comparing only users**		
In this study design, were analyzed the users and the results of them. The outcome measurements were 6-minute walk test, body mass, BMI, waist circumference and WHOQOL-BREF.		
**19) CONSORT: All important harms or unintended effects in each group**		
"No serious adverse events occurred during the study." No harms or unintended effects occurred in the groups.		
**19-i) Include privacy breaches, technical problems**		
No technical problems or privacy breaches occurred during the intervention.		
**19-ii) Include qualitative feedback from participants or observations from staff/researchers**		
This is not relevant in our article. Focus group interviews were performed but they are not described in this article. Feedback about the intervention is not included to this study as there was not developed any app.		
DISCUSSION		
**20) CONSORT: Trial limitations, addressing sources of potential bias, imprecision, multiplicity of analyses**		
**20-i) Typical limitations in ehealth trials**		
Limitations has been described. Large limitations were blindning and small sample size.		
**21) CONSORT: Generalisability (external validity, applicability) of the trial findings**		
**21-i) Generalizability to other populations**		
Generalizability has been discussed in the discussion.		
**21-ii) Discuss if there were elements in the RCT that would be different in a routine application setting**		
This study was a real-life study and did not develop any app.		
**22) CONSORT: Interpretation consistent with results, balancing benefits and harms, and considering other relevant evidence**		
**22-i) Restate study questions and summarize the answers suggested by the data, starting with primary outcomes and process outcomes (use)**		
Study question and main results have been discussed in the discussion.		
**22-ii) Highlight unanswered new questions, suggest future research**		
Future research need has been discussed in the discussion.		
Other information		
**23) CONSORT: Registration number and name of trial registry**		
Registration number has been introduced in abstract and introduction.		
**24) CONSORT: Where the full trial protocol can be accessed, if available**		
Trial protocol is not available. The trial registration can be seen in https://www.isrctn.com/ISRCTN61225589.		
**25) CONSORT: Sources of funding and other support (such as supply of drugs), role of funders**		
"This study was supported by the Social Insurance Institute of Finland."		
**X26-i) Comment on ethics committee approval**		
"The study was approved on October 15, 2015, by the Ethics Committee of the Central Finland Health Care District (Dnro: 12 U/2015)."		
**x26-ii) Outline informed consent procedures**		
The rehabilitees filled a consent in the rehabilitation center with the researchers. They had a chance to ask questions about the intervention. "After randomization, rehabilitees provided written consent for their participation in the intervention."		
**X26-iii) Safety and security procedures**		
The rehabilitees were guided to use technology in the rehabilitation center. In addition, they got information and guidance about cardiac rehabilitation. They had a team that included several healthcare professionals during the intervention.		
**X27-i) State the relation of the study team towards the system being evaluated**		
There were no conflict of interest.		
